# Cardiac diffusion-weighted MR imaging in recent, subacute and chronic myocardial infarction: a pilot study

**DOI:** 10.1186/1532-429X-13-S1-O46

**Published:** 2011-02-02

**Authors:** Jean-Pierre Laissy, Virginia Gaxotte, Nicoletta Pasi, Ahmed BenDriss, Laurent Feldman, P Gabriel Steg, Senior, Jean-Michel Serfaty

**Affiliations:** 1 Bichat University Hospital APHP, Paris, France

## Introduction

Delayed enhancement MR sequences (DE) are recognized as highly sensitive to detect recent and chronic myocardial infarction (MI) by visualizing contrast media accumulation in infarcted segments. Diffusion-weighted imaging (DWI) has recently been implemented in order to depict infarct-related myocardial edema and/or necrosis as a marker of acute but not chronic myocardial injury.

## Purpose

To investigate the clinical utility of DWI to detect recent MI and to differentiate it from subacute and chronic MI, with DE as reference.

## Methods

Seventy-four MI patients were studied in 3 groups. Group A included 34 recent (i.e. < 8 days) MI patients; group B, 22 patients with subacute (i.e. 9-90 days) MI; group C, 18 patients with chronic (> 90 days) MI; a fourth group (group D) included 24 control patients with no suspicion of MI and normal DE imaging. DWI (b, 250 to 500 sec/m^2^) and DE images of matched slices were acquired on a 1.5-T system. DWI and DE images were examined visually by 2 blinded observers for the presence or absence of hyperintense areas in corresponding segments. For infarct localization, DE images served as the reference standard.

## Results

The overall number of segments affected at DE was 272 (110 in recent, 94 in subacute and 68 in chronic MIs). Qualitative assessment of DWI compared to DE images per-patient yielded a sensitivity of 97% and a specificity of 61% to differentiate recent from chronic lesions, and a sensitivity of 97% and a specificity of 14% to diagnose recent from subacute MI. The relative apparent diffusion coefficient (ADC) or ADC ratio was significantly different between groups.

## Conclusions

DWI is a sensitive technique to diagnose recent MI. An imaging approach combining DWI and DE MR sequences accurately differentiates recent from chronic MI. From these preliminary results, one should expect DWI MRI to be used in the acute setting (in the triage of emergency patients with acute chest pain), to clarify if an MI is present or not in just a few minutes (making it better applicable in these patients as compared to DE MRI, as the latter technique requires more time).

